# A High-Isolation Optically Transparent 2 × 2 Antenna Array Using Metal Mesh Material

**DOI:** 10.3390/mi16050528

**Published:** 2025-04-29

**Authors:** Yufeng Yu, Yuanjia Dong, Yangyang He, Yi-Feng Cheng

**Affiliations:** 1 School of Information and Electrical Engineering, Hangzhou City University, Hangzhou 310015, China; yuyufeng.10@gmail.com; 2 School of Electronics and Information Engineering, Hangzhou Dianzi University, Hangzhou 310018, China; 231040039@hdu.edu.cn; 3 School of Microelectronics, Tianjin University, Tianjin 300072, China; heyangyang@tju.edu.cn; 4 State Key Laboratory of Millimeter Waves, Southeast University, Nanjing 210096, China

**Keywords:** optically transparent antenna, metal mesh, MIMO antenna, mutual coupling, shorting decoupling branches

## Abstract

This paper presents the design and implementation of a compact, high-isolation, optically transparent 2 × 2 MIMO antenna array. Optical transparency is achieved using a copper-based metal mesh material, which serves as both the radiating element and the ground plane, ensuring high radiation efficiency and minimal visual impact. The array consists of four monopole antennas, and mutual coupling is effectively suppressed through the integration of shorting decoupling branches and a common-ground structure. These techniques address both adjacent and diagonal (non-adjacent) coupling. A prototype was fabricated and experimentally validated. The experimental results demonstrate that the proposed antenna array achieves 20 dB isolation, 3.56 dB antenna gain, and 76% efficiency.

## 1. Introduction

Optically transparent antennas have garnered considerable attention due to their unique advantages, including enhanced aesthetics, compact size, and reduced weight. Transparent conductive oxide (TCO) films, widely used in transparent antenna design, offer a balance between optical transparency and electrical conductivity [1,2,3,4]. For instance, [1] presents an optically transparent reflection array at 28 GHz using an indium-doped tin oxide (ITO) substrate, while [2] introduces a dual-band transparent RFID antenna employing an Ag-coated polyester (AgHT) conductive layer. However, antennas based on TCO materials often suffer from low radiation efficiency due to high sheet resistance. Additionally, ITO films are fragile, limiting their suitability for mobile terminal applications. To overcome these challenges, multilayer films (MLFs) have been explored [3,4]. By stacking TCO layers above and below a conductive layer, MLFs enhance conductivity. Nevertheless, their relatively high sheet resistance (>2 Ω/sq) due to their thin structure remains a concern. Recently, metal mesh films (MMFs) have emerged as an alternative for transparent patch antennas [5,6,7], offering superior efficiency and stable radiation performance compared to MLFs.

Multi-input–multi-output (MIMO) technology is widely employed in wireless communication systems to enhance channel capacity [8,9,10,11,12,13,14]. However, integrating multiple antennas introduces strong mutual coupling, which degrades system performance. Various decoupling techniques have been proposed to mitigate this issue, including electromagnetic band-gap (EBG) structures [15] and defected ground structures (DGSs) [16], which suppress surface waves. Additionally, array-antenna decoupling surfaces [17] reduce spatial coupling, while neutralization lines [18] and parasitic elements [19] are commonly used to increase isolation. Decoupling matching networks (DMNs) [20,21] also effectively mitigate coupling. However, these approaches often require additional space and may introduce trade-offs, such as reduced bandwidth and altered radiation patterns.

While transparent antennas have been widely explored in single-antenna configurations, their extension to MIMO systems presents additional challenges. First, transparent conductive materials, such as transparent conductive oxides (TCOs) and metal mesh films (MMFs), exhibit higher sheet resistance than conventional metals, leading to increased ohmic losses and reduced radiation efficiency in multi-element designs. Second, strong mutual coupling between antenna elements, a common challenge in MIMO systems, becomes even more problematic in transparent MIMO antennas because high-resistance materials affect current flow and coupling paths differently compared to traditional conductors. Finally, many well-established MIMO decoupling techniques—such as EBG structures, defected ground structures (DGSs), and parasitic elements—often rely on additional conductive structures that may compromise optical transparency. These challenges necessitate the development of innovative transparent MIMO antenna designs that achieve high radiation efficiency, strong isolation, and excellent optical transparency.

To address these challenges, this article proposes a novel high-isolation optically transparent 2 × 2 MIMO antenna array. The design employs metal mesh material for both the antenna elements and the ground plane, ensuring optical transparency. Additionally, a simple shorting decoupling branch (SDB) structure is introduced to effectively suppress mutual coupling between antenna elements, achieving an isolation level below −20 dB. The proposed antenna array was fabricated and measured, demonstrating high isolation (20 dB) and high radiation efficiency (76%), providing a promising solution for future transparent MIMO antenna designs.

## 2. Geometry of the Proposed Antenna Array

**Figure 1:**
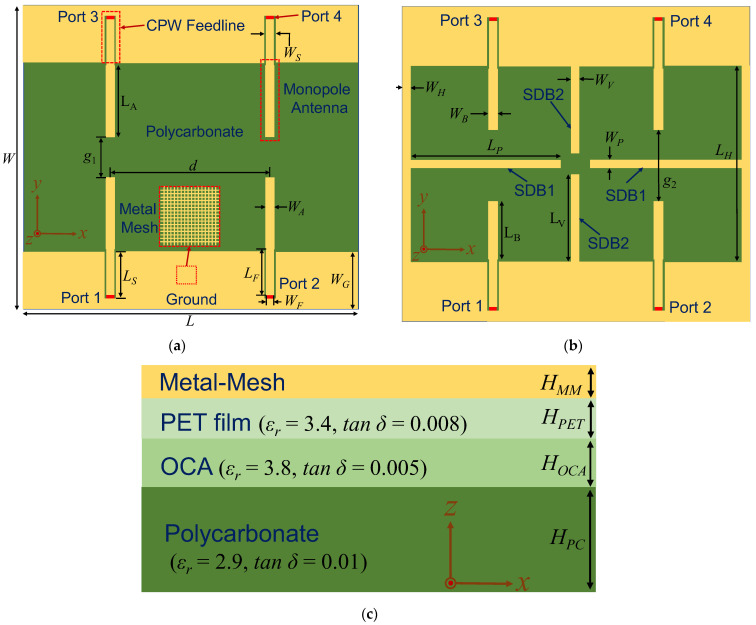
Geometry of the proposed optically transparent 2 × 2 MIMO antenna: (**a**) initial design; (**b**) proposed design; (**c**) Stacked distribution. The geometric parameters are listed as follows: *W* = 95 mm, *L* = 105 mm, *W_G_* = 18 mm, *W_A_* = 3.1 mm, *L_A_* = 22.5 mm, *W_S_* = 3.5 mm, *L_S_* = 14.9 mm, *W_F_* = 2.7 mm, *L_F_* = 14.5 mm, *W_B_* = 3 mm, *L_B_* = 16 mm, *W_H_* = 2 mm, *L_H_* = 59 mm, *W_V_* = 2 mm, *L_V_* = 22.8 mm, *W_P_* = 2 mm, *L_P_* = 45.8 mm, *H_MM_* = 3 um, *H_PET_* = 0.05 mm, *H_OCA_* = 0.1 mm, *H_PC_* = 1.5 mm, *d* = 50 mm, *g*_1_ = 12.8 mm, and *g*_2_ = 24.5 mm.

Figure 1 illustrates the initial and proposed designs of the optically transparent 2 × 2 MIMO antenna array. As shown in Figure 1a, each antenna element of the initial design consists of a monopole radiator and a coplanar waveguide (CPW) feedline. The four antenna elements are arranged in a centrally symmetric manner, with center-to-center spacing of *d* between adjacent elements. The use of a dense metal mesh structure ensures the optical transparency of the antenna array.

Figure 1b depicts the geometry of the proposed design. Decoupling is achieved by introducing shorting decoupling branches (SDBs) between the antenna elements along both the x- and y-directions and by implementing co-grounding between the upper and lower ground planes.

Figure 1c presents the stacked configuration of the proposed antenna array. The polycarbonate substrate, known for its excellent transparency, serves as the foundation. An optically clear adhesive (OCA) layer is deposited on top of the polycarbonate substrate, followed by a polyethylene terephthalate (PET) film layer and then a metal mesh layer. The OCA layer facilitates bonding between the PET film and the polycarbonate substrate, while the PET layer provides a suitable surface for metal mesh deposition. The radiators, ground, and SDBs are positioned on the top layer, ensuring a low-profile structure with minimal sheet resistance. Except for the metal mesh layer, all the other layers exhibit high optical transparency, preserving the overall transparency of the antenna array.

## 3. Results and Analysis of the Proposed Antenna Array

This section presents the simulation and experimental results of the proposed antenna array. We begin by examining the antenna’s evolution through three design stages and analyzing the mutual coupling behavior using S-parameters and electric field distributions. A parametric study is then performed to assess the influence of the structural variables on the decoupling performance. Finally, a fabricated prototype is measured to verify the simulated performance.

### 3.1. Evolution Designs and Decoupling Analysis

**Figure 2:**
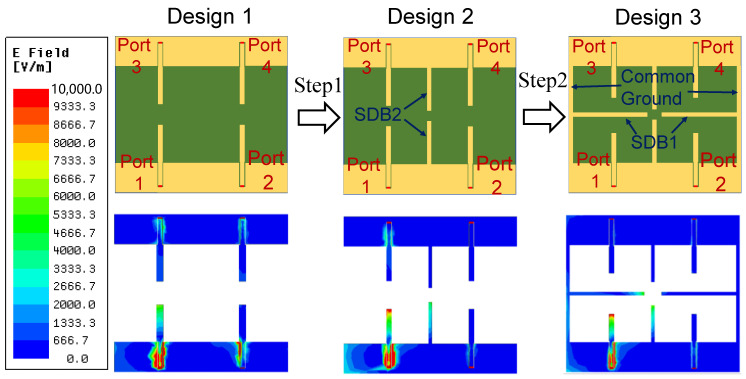
Evolution procedure of the proposed 2 × 2 MIMO antenna from Design 1 to Design 3 (proposed design) and the E-field distribution of each design with only port 1 excited.

Figure 2 illustrates the evolution process of the 2 × 2 MIMO antenna array and the corresponding electric field (E-field) distributions for each design. Due to the centrally symmetric arrangement of the four antenna ports, the analysis focuses on port 1 excitation, with the other ports terminated at 50 Ω loads.

**Figure 3:**
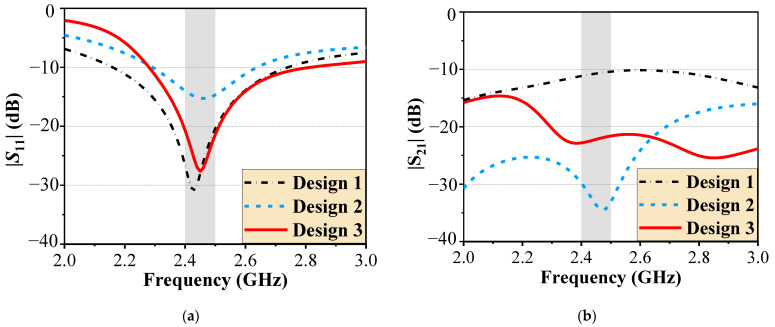

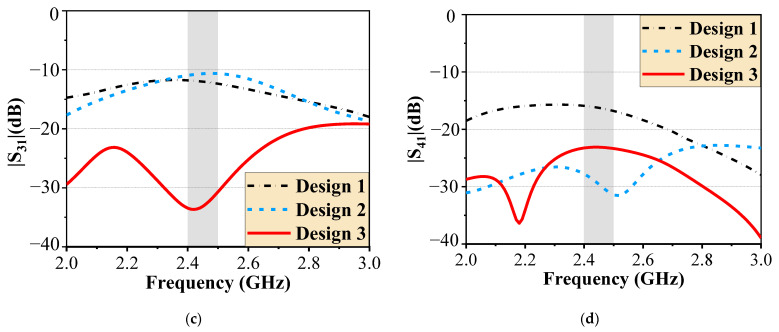
Simulated *S*-parameters of Designs 1, 2, and 3. (**a**) |S_11_|, (**b**) |S_21_|, (**c**) |S_31_|, and (**d**) |S_41_|. The grey areas correspond to the 2.4 GHz WLAN band.

Design 1 consists of a coupled array, where antenna 1 and antenna 2 share a common ground, while antenna 3 and antenna 4 share another ground. As shown in Figure 3a, the −10 dB bandwidth of Design 1 ranges from 2.21 GHz to 2.7 GHz, covering the 2.4 GHz WLAN band (2.4 GHz–2.48 GHz). However, the close proximity of the antenna elements results in significant mutual coupling, as indicated in Figure 3b–d, where the coupling coefficients |S_21_|, |S_31_|, and |S_41_| remain below only −11 dB.

To improve port isolation, Design 2 builds on Design 1 by incorporating SDB2 between antenna 1 and antenna 2 (similarly applied to antenna 3 and antenna 4). As shown in Figure 3, the antenna maintains its operational frequency band. Moreover, the coupling between horizontally adjacent elements (|S_21_|) and diagonal elements (|S_41_|) is significantly reduced to below −30 dB. However, the vertical coupling (|S_31_|) remains inadequately suppressed at approximately −10 dB.

To address the limitations of Design 2, the final design introduces a common ground configuration and incorporates SDB1, further enhancing isolation across all ports. Compared to Design 1, the isolation is improved by 10 dB (|S_21_|), 12 dB (|S_31_|), and 10 dB (|S_41_|).

Figure 2 also illustrates the E-field distribution for each design when only port 1 is excited, while the remaining ports are terminated with 50 Ω loads. In Design 1, significant mutual coupling is observed at ports 2, 3, and 4, primarily due to induced currents from antenna 1. After the first decoupling step, a notable reduction in the E-field strength is observed at ports 2 and 4. In Design 2, the E-field distribution indicates that the SDB2 exhibits a strong electric field. The original mutual coupling is reduced because SDB2 creates an additional coupling path between antennas that counteracts the original coupling path. As a result, after introducing SDB1 and the common ground in Design 3 (the proposed design), the E-field level at port 2 is also significantly reduced. This improvement is attributed to the introduction of additional coupling paths, which further enhance port isolation compared to Design 2.

### 3.2. Parametric Study

**Figure 4:**
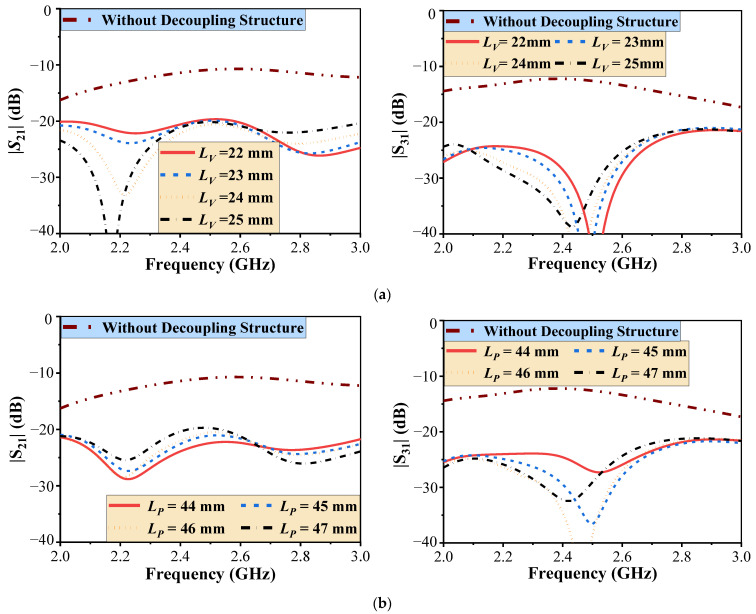
Simulated |S_21_| and |S_31_| versus the length of (**a**) SDB2 (*L_v_*) and (**b**) SDB1 (*L_p_*).

To further analyze the decoupling performance of the antenna, it is essential to investigate the role of the introduced shorting decoupling branches (SDBs). Figure 4a illustrates the impact of varying the length *L_v_* (the length of SDB2) on the primary coupling parameters, i.e., |S_21_| and |S_31_|. As *L_v_* increases, both decoupling zeros of |S_21_| shift toward lower frequencies. However, the isolation within the operating band (2.4–2.5 GHz) remains relatively stable. Similarly, the optimal decoupling frequency for |S_31_| also shifts toward lower frequencies as *L_v_* increases. Therefore, by adjusting *L_v_*, it is possible to tune the decoupling zero to align with the antenna’s center frequency, thereby achieving optimal isolation between port 1 and port 3.

Likewise, Figure 4b illustrates the effect of SDB1 on |S_21_| and |S_31_|. The length *L_p_* primarily influences the magnitude of the decoupling for |S_21_| without significantly affecting the frequency of the decoupling zero. In contrast, for |S_31_|, both the magnitude and frequency of the decoupling zero are influenced by *L_p_*. Notably, larger *L_p_* values cause the decoupling zero to shift toward lower frequencies.

In summary, the length of the decoupling strip plays a crucial role in the decoupling effect. By optimizing the decoupling strip length, the best decoupling performance can be achieved.

## 4. Experimental Verification

**Figure 5:**
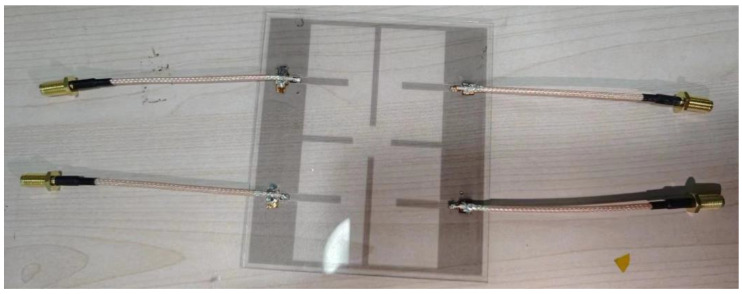
Photograph of the fabricated transparent 2 × 2 MIMO antenna arrays.

To validate the proposed high-isolation, optically transparent 2 × 2 MIMO antenna array, a prototype was fabricated and measured. Figure 5 shows the fabricated transparent antenna, where all four metallized antenna ports are connected using coaxial cables. The regions of the antenna requiring soldering were metallized in advance. This step is essential, as high-temperature welding could otherwise melt the PET film and compromise the integrity of the metal–mesh structure. 

**Figure 6:**
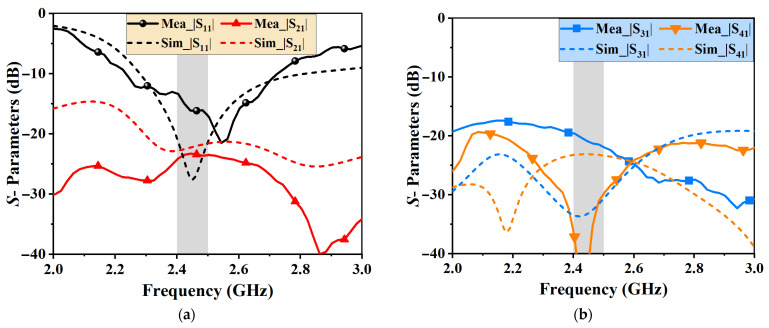
Measured and simulated S-parameters of the fabricated 2 × 2 MIMO antenna array. (**a**) |S_11_| and |S_21_|; (**b**) |S_31_| and |S_41_|. The grey areas correspond to the 2.4 GHz WLAN band.

The simulated and measured *S*-parameters are depicted in Figure 6. The measurement was conducted using a two-port vector network analyzer. The simulated and measured results exhibit good agreement, with minor discrepancies attributed to manufacturing tolerances and port metallization. Notably, the measured port isolations (|S_21_|, |S_31_| and |S_41_|) exceed 20 dB across the 2.4–2.5 GHz Wi-Fi band, with |S_31_| surpassing 30 dB.

**Figure 7:**
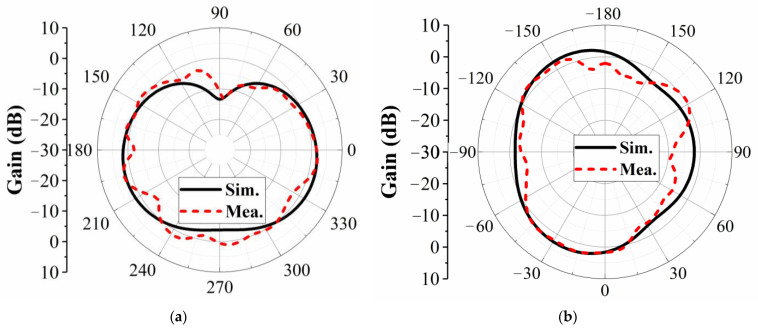
Measured and simulated radiation patterns of the proposed antenna at 2.45 GHz. (**a**) xoz-plane. (**b**) yoz-plane.

Figure 7 presents the simulated and measured radiation patterns of the proposed 2 × 2 MIMO antenna at 2.45 GHz in the *xoz*- and *yoz*-planes, showing relatively good agreement. The measurements were conducted in a far-field anechoic chamber. Notably, only port 1 was excited, while ports 2, 3, and 4 were terminated with 50 Ω matching loads.

The envelope correlation coefficient (ECC) is a critical metric for evaluating the radiation pattern diversity in MIMO systems. It is calculated based on measured far-field pattern data, where a lower ECC generally indicates higher channel capacity. In this study, the ECC values between Antenna 1 and Antenna 2 remain below 0.0502 within the 2.4–2.5 GHz frequency range. Additionally, the ECC between Antenna 1 and Antenna 3 is below 0.0268, while that between Antenna 1 and Antenna 4 is below 0.0116. The measured total efficiency exceeds 73.55% across the 2.4–2.5 GHz frequency band. Furthermore, the prototype antenna achieves a gain greater than 4.82 dB.

Table 1 provides a performance comparison with State-of-the-Art Transparent MIMO Antennas. In this work, the term “compact dimensions” refers to the center-to-center spacing between antenna elements, which is a commonly used metric to evaluate the physical size of MIMO antenna arrays. As summarized in Table 1, several existing two-element transparent MIMO antennas (e.g., [9,13]) achieve smaller spacing than our design. However, these configurations involve only a single mutual coupling path and are relatively simple to decouple. In contrast, the proposed design features a four-element MIMO configuration, requiring simultaneous suppression of multiple coupling paths. While its physical footprint is slightly larger than some other designs (e.g., [10]), it achieves significantly better isolation (>20 dB) and radiation efficiency (76%), thus offering a well-balanced trade-off between compactness and electromagnetic performance.

## 5. Conclusions

In this paper, we present a highly isolated, optically transparent 2 × 2 MIMO antenna array designed for Wi-Fi applications. The metal mesh technique ensures that both the radiators and ground plane are conductive while maintaining optical transparency. To mitigate coupling between horizontally adjacent antenna elements, parallel shorting decoupling branches (SDBs) are implemented. Additionally, to suppress coupling between vertically adjacent elements, a common ground configuration and perpendicular SDBs are introduced. By incorporating both parallel and perpendicular SDBs, the proposed design achieves port isolation exceeding 20 dB across all antenna elements. The antenna array demonstrates high isolation, adequate radiation efficiency, low sheet resistance, and excellent optical transparency, making it a promising candidate for wearable and conventional wireless communication devices.

## Figures and Tables

**Table 1:** Comparison with State-of-the-Art Transparent MIMO Antennas.

Ref. No. (Year)	Conductive Material	No. of Elements	Antenna Type	Frequency Band (GHz)	Center-to-Center Distance (mm)	Isolation (dB)	Gain (dB)	Efficiency
[9] (2017)	Metal Mesh	2	Meandered Monopole	2.4–2.48/ 5.15–5.8	0.18λ	15	0.74/ 2.3	40%
[10] (2021)	AgHT-4	4	Irregular Patch	2.2–6	0.34λ	15	0.5	41%
[11] (2020)	AgHT-8	4	Patch	24.1–27.2/ 33–44.1	1.02λ	16	2.9/ 3.1	75%
[12] (2021)	FTO and ITO	2	Monopole	2.4–11	0.44λ	20	2	60%
[13] (2023)	Metal Mesh	2	Patch	5.7–5.9	0.33λ	20	3.55	59%
This work	Metal Mesh	4	Monopole	2.4–2.5	0.41λ	20	3.56	76%

## Data Availability

The original contributions presented in this study are included in this article. Further inquiries can be directed to the corresponding author.
